# eQTL analysis: A bridge from genome to mechanism

**DOI:** 10.1016/j.gendis.2025.101850

**Published:** 2025-09-17

**Authors:** Zhe Jia, Jing Xu, Yingnan Ma, Siyu Wei, Chen Sun, Xingyu Chen, Jingxuan Kang, Haiyan Chen, Chen Zhang, Yu Dong, Junxian Tao, Xuying Guo, Hongchao Lv, Guoping Tang, Yongshuai Jiang, Mingming Zhang

**Affiliations:** a College of Bioinformatics Science and Technology, Harbin Medical University, Harbin, Heilongjiang 150086, China; b Department of Medical Engineering, The Fourth Affiliated Hospital of School of Medicine, and International School of Medicine, International Institutes of Medicine, Zhejiang University, Yiwu, Zhejiang 322000, China

**Keywords:** Expression quantitative trait locus, Gene expression, Genetic variation, Genome-wide association study, Single nucleotide polymorphism

## Abstract

Expression quantitative trait locus (eQTL) refers to a genetic variation associated with the expression of specific genes. It has been widely applied to explain the regulatory mechanisms linking genetic variations to complex traits or diseases. Several eQTLs have been identified from tissues and single cells in individuals. Furthermore, the integration of eQTL and other omics data can be used to detect novel susceptibility genes and consequently understand the dynamic regulation of trait-associated genetic variations at the system level. Here, we review the identification methods, analysis tools, research progress, and common data resources of eQTLs, as well as their role in four typical diseases. Finally, we discussed the application fields, challenges, and future development perspectives of eQTL.

## Introduction

One of the challenges faced by researchers in the post-genomic era is understanding the genetic mechanism between genomic variations (such as single-nucleotide polymorphisms or SNPs) and the phenotype. Quantitative trait loci (QTLs) are genomic loci that exert a quantitative effect on certain traits.^1^ Expression quantitative trait loci (eQTL), the most common QTLs, are regulatory genetic variation loci that govern the expression of specific genes. The standard method of identifying eQTLs is a linear regression model in which gene expression is considered a quantitative trait to quantify the effect of genetic variation on gene expression.^2^

Numerous studies have implicated eQTLs in multiple ways. For example, the eQTLs located within the gene transcription initiation regions have been demonstrated to affect the affinity of transcription factor binding sites or alter chromatin accessibility, thereby altering gene expression and functions. Similarly, the eQTLs located in the coding region of the gene directly influence the activity of the encoded protein.^3^ Another mode of action of eQTLs is to regulate the expression of target genes through regulatory proteins or microRNAs.^4^ However, the most important function of eQTLs is the explanation of the mechanism between trait-associated genetic variants in non-coding regions and complex traits after genome-wide association studies (GWAS). Several methods have been developed using this concept, such as the co-localization analysis. In addition, significantly associated SNPs in GWAS that have been detected as eQTLs are speculated to be functional genetic variations, but they need to be further verified. eQTLs can also be used to study causal inference. For example, transcriptome-wide association analysis (TWAS) uses eQTL data to predict gene expression and identify the association between expression and the associated trait.^5^ Mendelian randomization can infer causal genes regulated by eQTLs for a complex trait by integrating GWAS and the eQTL summary statistics.^6^ Initially, eQTL analysis was conducted using the gene microarray data. The development of sequencing technology has allowed eQTL researchers to use both bulk sequencing data and single-cell RNA sequencing (scRNA-seq) data. For instance, Will et al. (2013) demonstrated the impact of single-cell analysis on genetic variants.^7^ Several tissue-specific eQTLs and cell-type-specific eQTLs have been discovered. Numerous studies have reported that single-cell eQTL studies can be used to understand the complex genetic mechanisms of several diseases.^8^^,^^9^ For instance, Yazar et al identified 26,597 independent cis-eQTLs in 14 immune cells by integrating the scRNA-seq and genotype data from 1.27 million peripheral blood mononuclear cells from 982 healthy samples, and found that approximately 305 eQTLs were associated with autoimmune diseases because of altered gene expression in specific cell types.^10^ Nathan et al reported enriched autoimmune-related genetic variants in cell state-dependent eQTLs and demonstrated that cell state was crucial to explain the function of eQTLs.^11^ At present, molecular eQTL annotation has emerged as a powerful strategy to explain the genetic mechanisms of diseases.

Identification of eQTLs and elucidation of their functions in several diseases will assist us in better understanding the disease etiology and facilitate disease diagnosis or treatment. Here, we review the relevant knowledge of eQTLs, including their identification and analytical methods, development across different physiological periods, existing data resources, and their application in four typical diseases.

## Identification and analysis of eQTLs

eQTLs play an irreplaceable role in the pathogenesis of several diseases. It is indispensable to be familiar with eQTL data analyses and identification methods.^12^^,^^13^ Here, we systematically used the data for analysis, identification, analysis tools, and subsequent analysis for researchers.

### Data

The eQTL analysis requires multiple data during calculation, including SNP genotype matrix and gene expression matrix from the same samples, physical location data, and optional covariates matrix.

**Raw genotype data** are required to perform eQTL analysis. Briefly, genotype data are usually labeled as 0, 1, and 2, representing common allele homozygous, heterozygous genotype, and minor allele homozygous, respectively. In the case of insufficient genotype density in certain samples, genotype imputation is performed using HapMap^14^ (http://www.hapmap.org/) or 1000 Genomes Project^15^(http://www.1000genomes.org/) as the reference data using the IMPUTE2 software.^16^ The criteria for selecting SNPs after imputation are: i) minor allele frequency (MAF) ≥ 5%, ii) SNP missing rate <5%, and iii) Hardy–Weinberg equilibrium test *p*-value >1 × 10^−6^.^17^^,^^18^

**Gene expression data** can be microarray or sequencing data. These data are finally converted into a gene expression matrix. The R package “affy” can read raw microarray data and perform standardized processing workflows for it.^19^^,^^20^ Raw sequencing data need to be processed using multiple software tools for quality control, data filtering, normalization, and other steps to be converted into a matrix format.

**Covariate data** are processed into a matrix for better analysis, with columns representing samples and rows representing different covariates. Usually, apart from SNPs, other factors affecting gene expression are selected as covariates, and biologically relevant factors such as age, gender, and race should be prioritized. Other potential confounding factors need to be evaluated for their impact on gene expression to ensure the selection of the appropriate covariates.

**Location files** for genes and SNPs are required input for eQTL mapping so that the software knows which gene-SNP pairs to include for cis or trans eQTL testing. Gene positions are obtained from the National Center for Biotechnology Information’s (NCBI) genome database (www.ncbi.nlm.nih.gov/genome/), whereas SNP positions are obtained from the dbSNP database (https://www.ncbi.nlm.nih.gov/SNP/).

### Identification of eQTLs

**Figure 1:**
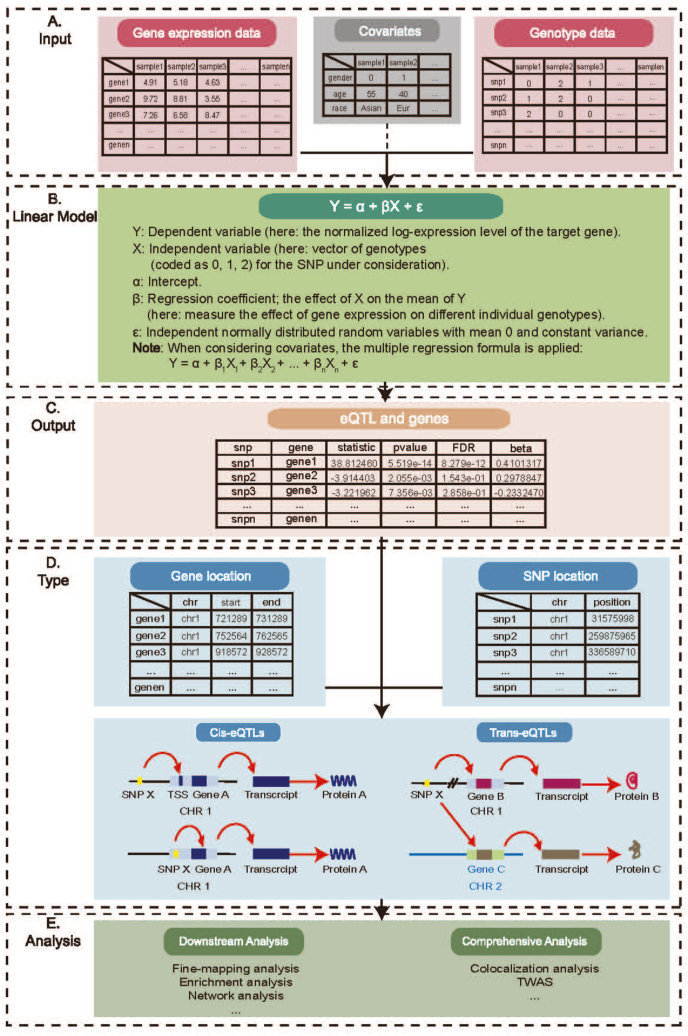
Workflow for eQTL identification and analysis. **(A)** Input data preparation. **(B)** Linear regression for eQTL identification. **(C)** Results of eQTLs. **(D)** Different types of eQTLs. **(E)** Subsequent analysis of eQTLs.

eQTL analysis, a linear regression analysis, includes the SNP genotype as the independent variable and gene expression as the dependent variable (Fig. 1A).^21^ For example, a SNP has two alleles (A and a), and $g_{i}$ is the expression value of the ith gene. The linear regression model of detecting eQTL is $g_{i}=\alpha_{i}+\beta_{i}x+\varepsilon_{i}$, where x represents the genotype, and AA, Aa, and aa can be coded as 2, 1, and 0, respectively (Fig. 1B). Multiple regression is used in cases including covariates. The linear regression model is fitted to detect eQTLs, and statistical data such as SNP–gene pairs, beta values, *p*-values, and distances between SNPs and genes can be obtained. Type I errors are controlled using commonly applied multiple testing correction methods, such as Bonferroni and false discovery rate. When the adjusted *p*-value is smaller than the usually set ɑ (*e.g.*, 0.05), a SNP is identified as an eQTL (Fig. 1C).

eQTLs are classified into cis-eQTLs and trans-eQTLs based on the distance between eQTLs and transcription start sites of regulated genes. cis-eQTLs refer to genetic variations located within a genomic window of the target gene, such as regions upstream and downstream of 1 Mb region.^22^^,^^23^ They are known to affect the structure of gene regulatory regions or chromatin and alter gene expression by influencing transcripts in the coding regions.^24^ In contrast, trans-eQTLs constitute genetic variations located outside this window region of the target gene, including those on different chromosomes, and can modify gene expression by interacting with regulatory proteins, such as transcription factors.^17^ For instance, the eQTL “SNP X” is shown in Figure 1D. The left image illustrates that SNP X is a cis-eQTL for gene *A* located on the same chromosome as the regulated gene *A*. Conversely, the right image displays that, being far from the regulatory gene, SNP X is a trans-eQTL for target genes *B* and *C*. Although both eQTL types can explain the regulatory relationship between genetic variations and gene expression, most present studies focus on the regulatory function of cis-eQTLs.^18^ Because cis-eQTLs are located near the target gene and typically exert stronger effects on gene expression, they are more likely to influence gene expression through direct regulation. cis-eQTLs simplify causal inference from genetic variation to phenotype, facilitating subsequent functional studies and drug target discovery. In contrast, trans-eQTLs often involve indirect regulatory networks, making them more challenging to study, and yet remain indispensable for comprehensively understanding genetic regulation.

We conducted a comprehensive eQTL analysis on simulation data using the MatrixeQTL software; specific details are provided in the supplementary material.

### Common eQTL identification tools

The increasing use of eQTL analysis has led to the application of numerous tools in this field.

**MatrixeQTL** (www.bios.unc.edu/research/genomic_software/Matrix_eQTL/) is a classic and common eQTL analysis software used for identifying cis-eQTLs and trans-eQTLs.^21^ It can efficiently analyze large-scale data, support linear model analysis of genotypes and expression levels, support the adjustment of multiple covariates (such as age and gender), and provide an R language interface. Its drawback is that the correction of multiple tests relies on traditional methods (such as Bonferroni), which may be overly strict. Additionally, it is limited in the analysis of single-cell data rand complex cellular heterogeneity.

**FastQTL** (http://fastqtl.sourceforge.net/) is another commonly used software, specifically designed for cis-eQTL analysis and widely applied in the analysis of projects such as Genotype-Tissue Expression (GTEx).^25^ Its advantages are fast running speed, support for covariate adjustment and normalization of gene expression data, optimized multiple corrections, and suitability for parallel analysis of large-scale single-cell datasets. Its disadvantage is that the ability to analyze trans-eQTL is relatively weak.

**QTLtools** (https://qtltools.github.io/qtltools/) applies to a variety of molecular QTL analyses, including eQTL, splicing QTL, and chromatin interaction QTL analysis. It supports multiple types of QTLs and phenotypic data (*e.g.*, RNA-seq, ATAC-seq), but the installation is relatively complex, has high requirements for computing resources, and requires a high-performance computing environment.^26^

In recent years, the rapid development of single-cell sequencing (scRNA-seq) technology has provided us with an opportunity to uncover associations at the single-cell level between genetic variations and gene expression. Many eQTL tools at the cellular level that have emerged. SCeQTL^27^ is an R package (https://github.com/XuegongLab/SCeQTL/), that can not only perform eQTL analysis on single-cell data, but also detect variations in gene expression associated with other grouping factors, such as cell lineages or cell types. It can capture cellular heterogeneity and reveal the regulatory effects masked by traditional batch analysis. However, currently, due to high data sparsity and low statistical power, a large sample size is required for its support. The analysis process is not standardized yet, and the computational complexity is high.

Single-cell eQTL analysis usually uses genetic variation and expression data from the same cells. However, the current technology of such paired data in single cells is still immature. The eQTLsingle method (https://github.com/horsedayday/eQTLsingle) addresses the limitation, which can detect mutations in coding regions using scRNA-seq data to capture both mutation and expression profiles from individual cells.^28^ Therefore, eQTLsingle makes it possible by identifying eQTLs only with scRNA-seq data or snRNA-seq data. Nevertheless, this approach has limitations in identifying potential eQTLs in non-coding regions and genes with low expression levels.

SURGE (https://github.com/BennyStrobes/surge) is a method to identify context-specific eQTLs from single-cell transcriptomic data, which can apply to any eQTL data.^29^ SURGE is particularly effective at automatically detecting latent contexts and demonstrates high statistical power. Nonetheless, it might face challenges in interpreting latent contexts, detecting small-effect eQTLs, and scaling to very large datasets.

Cell Regulatory Map (CellRegMap, https://github.com/limix/CellRegMap) is a statistical framework to perform multi-context eQTL mapping using scRNA-seq data based on a linear mixed model.^30^ CellRegMap can account for sample structure, including population structure and repeated observations from the same samples. Its key strengths include effective integration of diverse data types and the capability to uncover cell type-specific regulatory mechanisms. However, it necessitates high-quality multimodal data and may encounter challenges in interpreting complex regulatory networks.

Presently, there is a rising trend in extensive scRNA-seq and cell type-specific eQTL (ct-eQTL) investigations.^31^ It is worth noting the web tool developed by Igor Mandric et al (https://mandricigor.github.io/ct-eqtl-design/) that effectively aids scientists in the optimization of ct-eQTL research experiment design.^32^ By entering desired cell types, budget constraints, and sequencing expenses, the optimal sample size and number of cells are automatically determined by this calculator, leading to a significant enhancement in statistical power.

Additionally, single-nucleotide variants can function as eQTLs. The RBP-Var tool (www.rbp-var.biols.ac.cn) is employed to investigate the relationship between single-nucleotide variants and gene expression, as well as provide annotations for functional variants involved in post-transcriptional regulation. The details of the aforementioned tools are available on our website (http://www.onethird-lab.com/eQTLbrowser).

### Analysis of eQTLs

The identified eQTLs can be mapped to the reference genome, with a few located within the genes and others located between genes. These eQTLs offer diverse analytical applications, including downstream analysis of regulatory relationships between the target gene and other genomic elements.^24^ For instance, enrichment analysis can deduce the biological functions associated with eQTLs, whereas network analysis investigates their impacts within a multi-omics framework.^33^ In addition to downstream analysis, integrated analysis is widely applied. GWAS, developed over a decade ago, is a well-established technique for identifying associations between SNPs and complex traits.^34^ Data sources such as UKbiobank^35^(https://www.ukbiobank.ac.uk/), GWAS catalog^36^, ^37^, ^38^ (http://www.ebi.ac.uk/gwas/), and GWAS ATLAS^39^ (https://bigd.big.ac.cn/gwas/) provide successful GWAS outcomes. Although hundreds of risk SNPs have been identified using GWAS,^40^ the mechanism between these SNPs and the complex traits remains elusive. In this regard, the eQTL analysis can detect the regulatory relationship between SNPs and gene expression and explain the regulation route from genetic variations to disease risk (SNP → gene expression → disease).^41^^,^^42^ Therefore, causal relationships can be studied by integrating eQTL and GWAS analyses.

It is noteworthy to mention TWAS, a method that integrates eQTL and GWAS to unravel the genetic regulatory mechanisms underlying complex traits or diseases.^5^ Its core principle involves constructing predictive models of gene expression to translate associations between genetic variants and phenotypes into associations between gene expression levels and phenotypes, thereby identifying potential causal genes. A variety of methods or software (*e.g.*, PrediXcan, S-prediXcan, FUSION, UTMOST) have been developed for TWAS analysis.^43^ TWAS, as a gene-based analysis method, presents a reduced multiple testing burden compared with GWAS. By presenting results at the level of specific genes, it facilitates subsequent functional analyses and enables more direct interpretation of biological significance. The GTEx project provides an exceptionally rich resource of genomic and transcriptomic data for TWAS analysis, allowing researchers to construct gene expression prediction models using this reference panel. TWAS is particularly well-suited for identifying susceptibility genes for complex diseases, as well as applications in early diagnosis, disease prediction, and risk assessment research.^44^ TWAS integrated with eQTL analysis has become a pivotal tool for dissecting the genetic architecture of complex traits by bridging genetic variation and functional genes. With continuous optimization of multi-tissue modeling, fine-mapping, and cross-omics integration methods, its applications have expanded from disease mechanisms to agricultural breeding. It holds great promise for future roles in precision medicine and molecular design breeding. However, challenges, such as cross-population data compatibility, dynamic regulatory mechanism resolution, and causal validation, remain critical bottlenecks requiring breakthroughs.

### Common eQTL analysis tools

Recently, several analytical tools have been applied for integrating eQTL and GWAS, facilitating further screening of disease-related functions of SNPs. Here, we introduced certain efficient and typical tools such as eCAVIAR (http://zarlab.cs.ucla.edu/tag/ecaviar/),^42^ SMR (https://yanglab.westlake.edu.cn/software/smr),^45^ XGR (https://xgr.r-forge.r-project.org/),^46^ and OmicKriging (http://www.scandb.org/newinterface/tools/OmicKriging.html).^47^ eCAVIAR is a Bayesian probabilistic model used to identify causal variants in both GWAS and eQTL studies based on the co-localization method. Its operating speed is fast. Summary-based Mendelian randomization (SMR) is a linear regression-based eQTL analysis tool known for its efficient processing speed. It facilitates simultaneous analysis of associations between gene expression and genotype data. XGR is a comprehensive bioinformatics toolkit offering several analysis modules, including eQTL analysis. The actual running speed could vary depending on the selected module and dataset size. OmicKriging is an eQTL analysis tool that utilizes Kriging interpolation; it has a slightly slower running speed, particularly when dealing with large-scale datasets. eCAVIAR and SMR can accept genotype and expression data in the summary statistics format as inputs, whereas OmicKriging specifically accepts data in the PLINK format. XGR, in contrast, supports data analysis in both VCF and PLINK formats. SMR provides exceptionally beautiful visualizations of the output data. Similarly, XGR can visualize the results. The performance of eCAVIAR is assessed from an algorithmic standpoint by comparing it against simulated data or known results. Key evaluation metrics include accuracy, recall, and F1 score. The efficacy of SMR is measured by comparing it with the established results. Relevant evaluation metrics include the precision of association statistics and *p*-values. Similarly, the performance of XGR is assessed by comparing it with known results. Common evaluation metrics include the accuracy of association statistics, *p*-values, and annotation information accuracy. The performance of OmicKriging is assessed by comparing it with known results. Common evaluation metrics include the accuracy of eQTL mapping and gene expression prediction. The details of all tools are available at http://www.onethird-lab.com/eQTLbrowser.

## eQTL in different development periods

**Figure 2:**
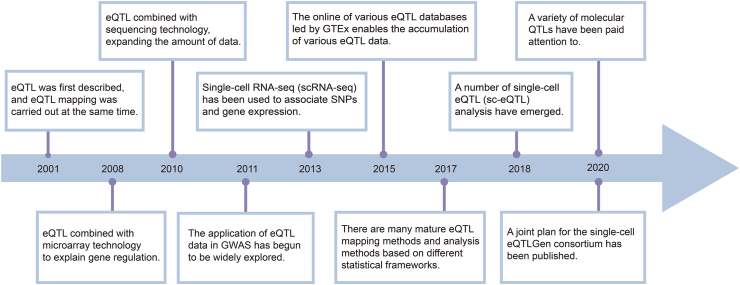
eQTL development.

The eQTL mapping analysis was first implemented in 2001, associating the SNPs with the target transcript abundance.^48^ Over the years, eQTL studies have evolved and progressed significantly (Fig. 2), driven by advancements in technology and methodologies. We will discuss the major developments in eQTL analysis across four periods: microarray period, high-throughput sequencing period, post-genome period, and single-cell sequencing period.

### Microarray period

In the 1990s, high-throughput DNA chips or microarrays were applied to different kinds of gene expression scenarios.^49^ These have emerged as efficient methods that can simultaneously perform gene expression analysis and genotyping.^50^ This became a prerequisite for the development of eQTL research. After obtaining gene expression and the SNP genotype of the same sample from the microarray, an eQTL is identified by regression analysis. Chip technology was used in the early study of eQTLs in several diseases, such as rheumatoid arthritis (RA) and ischemic stroke. eQTL analysis using Illumina BeadChip microarrays from 344 patients with RA revealed that *METL21B*, *JAZF1*, *IKZF3*, and *PADI4* were regulated by cis-eQTLs in CD4^+^ lymphocyte subsets, and alterations of their expression suggested a role of CD4^+^ lymphocyte subsets in an RA-specific effect.^51^ Four SNP-gene pairs were identified by microarray eQTL analysis in ischemic stroke, which were implicated in immune responses and contributed to the development of personalized treatment.^52^

Altogether, chip technology contributes to the development of early eQTL research and prompts researchers to understand the regulation of several genes from the perspective of genetic variations.^53^

### High-throughput sequencing period

The continuous advancement and evolution of experimental technology have led to the emergence of large-scale and high-resolution RNA-seq, greatly promoting the development of eQTLs and enhancing the understanding of eQTLs' potential regulatory mechanisms. Compared with the microarray technology, RNA-seq technology can more accurately explain the relationship between genetic variations and phenotypes. Therefore, RNA-seq technology has been widely used for eQTL research since 2010.^54^

In the era of high-throughput sequencing, eQTL analysis has greatly promoted the study of the pathogenesis of several diseases.^55^ For instance, an eQTL analysis using sequencing technology confirmed certain novel loci related to obesity, such as *Lpl*, *Lactb*, and *Ppm1l*.^56^ Another eQTL study of the prostate tissue revealed that rs13215402 regulated the expression of *RGS17*, which was finally confirmed as a susceptibility gene for prostate cancer. Specifically, the higher expression of *RGS17* is associated with the severity of prostate cancer.^57^

Several databases have emerged during the high-throughput sequencing period to store the eQTL results. The largest one is the GTEx database, which is a landmark and provides researchers with rich eQTL data to explore gene expression patterns in different tissues.^58^ Several eQTL analysis tools based on sequencing data have been developed, such as FastQTL^25^ and QTLtools.^26^

Although hundreds of eQTLs associated with complex traits have been identified, it is challenging to integrate eQTLs with other omics data for a comprehensive understanding of human diseases.

### Post-genome period

In the post-genome period, several methods have been applied to integrate the eQTL analysis with GWAS for the causal analysis of human diseases,^59^ such as SMR and co-localization. SMR-based integrative analysis has identified numerous risk genes in several diseases, such as *TRAF1* and *ANKRD55* in RA and *SNX19* and *NMRAL* in schizophrenia (SZ).^45^ For instance, Huang et al identified that inflammatory bowel disease-related variants had significant co-localization signals with eQTLs in CD4^+^ T lymphocytes.^60^ Similarly, Franceschini et al identified two candidate genes, namely, *CCDC71L* and *PRKAR2B*, by using GWAS of cardiovascular signatures to colocalize with eQTLs in the vascular tissue, leading to atherosclerosis in cardiovascular disease.^61^

The integration of eQTL and survival analyses for identifying survival-related eQTL has also been attempted in the study of different cancers. For example, Gong et al identified 22,212 survival-related eQTLs to explain the relationship between genetic variations and survival.^62^ Network analysis of integrating eQTL and other omics data is a current research hotspot.^63^ Mo et al constructed a genome-wide long non-coding RNA (lncRNA)-eQTL network and identified eQTL as an intermediary factor in mRNA–lncRNA functional modules.^64^

### Single-cell sequencing period

Traditional studies involving bulk RNA-seq data often do not capture information regarding allele-specific expression that pertains to specific cell types. The introduction of scRNA-seq around the mid-2010s has brought about new possibilities for analyzing eQTLs at the individual cell level, providing valuable insights into the diversity of cells, differentiation among cell types, and disease mechanisms.^65^ Early approaches for linking SNPs with gene expression in scRNA-seq data began to surface in 2013, establishing the groundwork for studying single-cell eQTLs.^7^ In 2018, the first genuine single-cell eQTL (sc-eQTL) analysis was conducted by Kang et al using droplet-based single-cell sequencing technology. They examined 78,000 peripheral blood mononuclear cells from eight major immune cell populations from 23 donors and identified 32 cis-eQTLs, 22 of which were specific to particular cell types.^66^ In the same year, Van Derwijst et al analyzed scRNA-seq data from 25,000 peripheral blood mononuclear cells of 45 Dutch donors and identified cell type-specific cis-eQTLs and co-expression QTLs.^67^

The rapid advancement of scRNA-seq technology has enabled numerous research groups to explore the intricate regulatory landscape in various biological systems, including peripheral blood mononuclear cells, T cells, induced pluripotent stem cells, brain cortex, and dopaminergic neuron differentiation.^8^ These investigations have yielded significant findings regarding cell-type-specific eQTLs associated with immune response genes,^10^^,^^68^^,^^69^ the genetic influence on gene expression during cellular reprogramming and differentiation processes,^70^ genetic regulatory mechanisms governing gene expression in various cell types like neurons, astrocytes, and oligodendrocytes, the genetic foundation of cellular diversity and functional specialization in the brain,^71^^,^^72^ and genetic factors that impact gene expression patterns in dopaminergic neurons.^73^ Thus, these discoveries enhance our comprehension of complex diseases (*e.g.*, autoimmune diseases and neurodevelopmental disorders), cellular reprogramming, and brain development, laying the groundwork for precision medicine and therapeutic interventions. Alongside sc-eQTL studies, several tools and approaches have been developed to optimize and design sc-eQTL analysis, including SCeQTL^27^ and scPower.^74^ Additionally, various resources are accessible, such as the single-cell eQTLGen consortium^75^ and the OneK1K cohort.^10^

Despite significant progress, several challenges and issues need to be addressed in sc-eQTL studies.^76^ The current analysis methods and tools require improvements to handle the complexity and unique features of single-cell data. Breakthroughs in sc-eQTL research are expected with further technological advancements. These breakthroughs will help unravel gene regulatory networks, uncover disease mechanisms, and support personalized medicine and therapeutic interventions.

## Common eQTL data resources

The innovation of sequencing technology and the wide application of eQTL identification methods have resulted in the development of certain large eQTL databases, providing great convenience for users to understand the functions of genetic variations in the genome.

**GTEx (V8)** (https://gtexportal.org/home/) is a comprehensive public resource, containing the eQTL data of 49 non-disease tissues from nearly 1,000 individuals.^77^ Data can be downloaded by tissue and eQTL type (cis-eQTL or trans-eQTL) from the GTEx database.^78^ GTEx contributes to the identification of disease-related risk genes. For example, Yang et al confirmed that *GLT8D1* and *CSNK2B* were risk genes for SZ using brain tissue data of 92 subjects from the GTEx database,^79^ thus providing novel ideas for the prevention and clinical treatment of SZ. Kundu et al identified 340 pathogenic variant loci with a high posterior probability for multiple immune-mediated diseases using the GTEx database.^80^

**eQTLGen** (www.eqtlgen.org/) is a free eQTL database that stores cis- and trans-eQTLs from 31,684 whole blood samples.^81^ In addition, the database stores single-cell eQTLs from more than 30,000 blood samples, contributing to differences in eQTLs' replication rates between single-cell data and mixed cell-type data.^18^ These data assist in understanding the tissue-specific expression of eQTLs in blood. A systematic study of pancreatic cancer identified rs3802266 as an important cis-eQTL for *ZHX2* using eQTLGen and found that the lower *ZHX2* expression was related to a higher risk of pancreatic cancer in individuals.^82^ In 2021, Yang et al identified SNPs, namely, rs13428812, rs116555563, and rs9977672, in multiple sclerosis and regulatory pathways shared between different diseases through a meta-analysis combined with eQTLGen.^83^ Multiple sclerosis and inflammatory bowel disease share the same tissue-specific heritability enrichment pattern for eQTLs in whole blood tissue, suggesting a biological basis for co-morbidity between the two.

**eQTL Catalogue** (www.ebi.ac.uk/eqtl) is an invaluable resource providing uniformly processed gene expression cis-eQTLs and splicing QTLs from 32 different studies, where summary statistics are available for several cell types and tissues.^8^ It provides a comprehensive collection of genetic variants associated with altered gene expression and splicing, and can systematically explain human GWAS associations across a wide range of cell types and tissues.^84^ Koel et al performed the GWAS meta-analysis, shedding light on the genetics of cervical cancer phenotypes and providing valuable information for risk stratification of cervical cancer.^85^ Chun et al utilized the data from the eQTL Catalog to identify pleiotropic variants impacting both gene expression and obstructive sleep apnea, thereby providing valuable insights into the underlying mechanisms of complex human diseases.^86^ eQTL Catalogue was updated in 2023, which increased the number of uniformly processed studies from 21 to 31 and added X chromosome QTLs for 19 compatible studies.^87^

**OneK1K** (www.onek1k.org/) is a large cohort containing scRNA-seq data from 1.27 million peripheral blood mononuclear cells collected from 982 donors.^10^ An eQTL analysis was performed across 14 immune cell types that identified 26,597 cis-eQTLs. The majority of these eQTLs exhibited cell type-specific effects.^8^ These findings contribute to our understanding of how genetic variation impacts gene regulation and signaling pathways in a cell type-specific manner. Yazar et al used the OneK1K to identify single-cell eQTLs in nine immune-mediated diseases and found that most eQTLs affected T-cell activation and signaling.^88^ Another study conducted using OneK1K confirmed the dynamic nature of HLA eQTL effects across different immune cells. This highlights the significance of including cell types when investigating the genetic mechanisms underlying HLA expression.^89^

**scQTLbase** (http://bioinfo.szbl.ac.cn/scQTLbase) is the first integrated comprehensive database designed to explore human sc-eQTLs, which unravel the genetic basis of cell type-specific gene regulation.^90^ By consolidating 304 sc-eQTLs datasets from diverse sources, scQTLbase spans 57 cell types and 95 cell states. It includes approximately 16 million SNPs significantly linked to cell type/state-dependent gene expression, along with ∼690,000 disease-associated sc-eQTLs derived from 3333 traits/diseases. Beyond sc-eQTL search and browsing functionalities, scQTLbase enables gene expression visualization in UMAP plots and colocalization visualization with GWAS datasets. scQTLbase will help in the discovery of disease susceptibility genes.

**SingleQ** (http://www.sqraolab.com/scqtl) is a single-cell eQTL interactive database, which collects sc-eQTL datasets and provides online visualization of sc-eQTLs across different cell types in a user-friendly manner.^91^ SingleQ sc-eQTL database curated up to 77467 eQTL summary statistics from 273 unique cell types covering different developmental stages of diverse tissues or cell states. SingleQ is the most comprehensive summary statistics by far.

**PancanQTL** (http://gong_lab.hzau.edu.cn/PancanQTL/) is the first cancer-related database that stores eQTLs of 9196 tumor samples from 33 cancer types in the Cancer Genome Atlas (TCGA). It contains 5,606,570 cis-eQTLs, 231,210 trans-eQTLs, 22,212 survival-related eQTLs, and 337,131 GWAS-related eQTLs, and supports querying, browsing, and downloading data.^62^ Carrot-Zhang et al^92^ identified tens of thousands of cancer-type-specific eQTLs using PancanQTL and explained the impact of reproductive system genetic variations on cancer gene expression. PancanQTLv2.0 (https://hanlaboratory.com/PancanQTLv2/) has updated the fine-mapping of causal variants for eQTLs, identifying eQTLs associated with drug response and immune infiltration. The database finalized 58,747 fine-mapped eQTL credible sets and identified 84,592,135 linkage associations between eQTLs and existing GWAS loci, marking a 50-fold increase compared with PancanQTLv1.0. Additionally, 659,516 associations between eQTLs and drug responses and 146,948 associations between eQTLs and immune cell abundance were contained in PancanQTLv2.0, which provides a foundation for the potential clinical application of eQTLs in cancer therapy.^93^

Several other eQTL databases, such as Blood eQTL Browser,^94^ EyeGEx,^95^ and ImmuNexUT,^96^ have been widely used. More details of these important eQTL databases are shown in Tables S1–S3.

## The role of eQTL in clinical diseases

eQTLs have several unique functions in disease research; they act as a bridge from genetic variations to functions and can cover the limitations of GWAS. Numerous disease-associated eQTL genes have been discovered, thereby elucidating the regulatory mechanism of risk SNPs and diseases from a functional perspective. We will focus on the contribution of eQTL analysis in four typical diseases: RA, type 2 diabetes (T2D), breast cancer, and SZ. Certain eQTL-gene pairs of four diseases in the whole blood are shown in Table S4. More details are available at www.onethird-lab.com/eQTLbrowser.

### Rheumatoid arthritis

RA is a chronic and inflammatory autoimmune disease that could lead to joint damage and systemic complications.^97^ It is known to affect approximately 0.5%–1% of the world’s population.^98^ The etiology of RA is complex, and genetic factors can explain approximately 60% of RA susceptibility.^99^ The eQTL analysis has provided a boost to understanding the pathogenic mechanisms underlying RA.

Recently, an eQTL analysis identified certain novel susceptibility genes and revealed a new genetic mechanism for RA. Thalayasingam et al identified novel eQTLs regulating *METL21B*, *JAZF1*, *IKZF3*, *PTPN22*, and *PADI4* in CD4^+^ T cells for the first time.^51^^,^^100^ Among these, SNP rs142845557 is located in a non-coding region, which is a synovial tissue-specific eQTL. It regulates chromatin accessibility in synovial tissue (such as the open state of enhancers or promoters), thereby influencing the expression of *JAZF1*. *JAZF1* plays a critical role in the activation of synovial fibroblasts and may be involved in joint inflammation and tissue destruction processes.^101^ *PTPN22* expression showed significant associations with RA risk across 44 tissues based on an integrative analysis of cross-tissue TWAS and eQTL. The mechanistic investigation revealed that the rs2476601 modulated *PTPN22* expression and regulated T-cell receptor signaling pathway activity. Dysfunctional *PTPN22* led to T-cell overactivation, disrupted immune tolerance, and thereby promoted autoantibody production in RA pathogenesis.^102^ An example in *PADI4* demonstrates that eQTL analysis can help us understand disease risk from genotype to RA. The expression level of *PADI4* is associated with rs2240335. The A allele at rs2240335 is associated with an elevated risk of RA. rs2240335-A is associated with increased expression of *PADI4* in neutrophils but reduced expression in monocytes.^103^ Ishigaki et al identified that an eQTL rs7616215 regulated *CCR2* and *CCR3* simultaneously in monocytes, thereby activating immune conditions and increasing the risk of RA.^104^ These results highlight how eQTLs serve as functional bridges connecting genetic variants to biological mechanisms.

Single-cell eQTL modeling is an increasingly popular research direction, and environment-dependent effects in RA pathogenesis have been comprehensively studied using single-cell analyses. In total, 6511 *cis*-eQTLs were identified by Nathan et al, among which RA risk variants near *ORMDL3* and *CTLA4* were enriched in cell-state-dependent eQTLs.^11^ Regulatory regions of immune cells in RA tissues overlap with several GWAS gene loci, especially CD4^+^ T cells and CD8^+^ T cells, which is consistent with earlier findings.^105^

### Type 2 diabetes

T2D is a common metabolic disease affecting more than 300 million people worldwide.^106^ Although GWAS has identified several susceptibility SNPs in T2D, it does not completely explain how risk loci lead to T2D, and eQTLs fills this gap well. Here, we explored the relationship between genetic variants and risk genes influencing T2D susceptibility and associated glycemic characteristics.

Certain effects on islet cell function have been identified through various studies involving eQTLs. For example, the typical eQTL rs4547172, as identified by Keildson et al, leads to the overexpression of the *PFKM* gene, which is associated with fasting plasma insulin, reduced metabolic flexibility, and the promotion of T2D.^107^ The presence of the cis-eQTL rs75409866 of the *KCNA6* gene in pancreatic islets, as confirmed by Varshney et al, impacts glucose-stimulated insulin secretion (*GSIS*) and contributes to the development of T2D.^108^ Khamis et al reported that eQTLs regulated *TTLL6* (rs11657371), *MLX* (rs646123), and *KIF9* (rs2276854) through co-localization with T2D GWAS, even though these were not considered risk loci in GWAS, they still exhibited significant associations with T2D risk genes.^109^ Mulder reported that the cis-eQTL rs950994-A of *TFB1M* enhances TFB1M expression, thus inhibiting insulin secretion in human islets, making individuals more susceptible to T2D.^110^^,^^111^ Additionally, co-localization analyses have yielded new insights into the mechanisms underlying T2D. Broadaway et al identified 36 insulin-promoting genes that co-localize with pancreatic islet eQTLs, potentially identifying candidate genes for the disease.^112^ A co-analysis with depression revealed a significant causal effect on T2D. CorrespondingeQTLs for *CCND2* and *IRS1* were found in pancreatic islets, brain, and fat, indicating the multi-tissue and pleiotropic nature of T2D pathogenesis. Furthermore, Mendelian randomization demonstrated causal effects, emphasizing the importance of preventing T2D when depressive symptoms first appear.^113^ These results not only elucidate the genetic architecture underlying T2D but also provide valuable insights into the molecular mechanisms driving disease progression, emphasizing the importance of eQTLs as a bridge from genomic variation to functional consequences in complex metabolic diseases.

### Breast cancer

Breast cancer is the most common cancer in females worldwide,^114^ which presents familial aggregation, suggesting that genetics is an important factor. GWAS and meta-analysis have identified several risk loci, such as *BRCA1*, *BRCA2*, and *PALB2*; however, these loci can only explain about 30% of the familial risk.^115^ The eQTL analysis can directly or indirectly probe the expression patterns affecting target genes and thus influence breast cancer, largely compensating for heritability analysis methods.

Li et al performed a trans-eQTL analysis using breast cancer data from TCGA and identified three new risk loci, namely, rs2046210, rs418269, and rs471467, regulating *ESR1*, *MYC*, and *KLF4*,^116^ which can promote the occurrence and development of breast cancer from multiple signaling pathways and transcriptional regulatory mechanisms.^115^^,^^117^^,^^118^ In addition, regulatory elements of breast cancer target genes are affected by eQTLs. Guo et al found that cis-eQTLs, including rs11552449 (*DCLRE1B*), rs7257932 (*SSBP4*), rs2236007 (*PAX9*), and rs73134739 (*ATG10*), significantly altered the promoter activity of these breast cancer-related target genes.^119^ Among them, rs11552449 can affect the expression of *DCLRE1B* by enhancing its promoter activity, thereby affecting DNA stability and increasing the probability of cancer.^119^ Similarly, rs2236007 can promote the occurrence and development of breast cancer by regulating *PAX9*, changing the transcriptional activity of downstream genes, and participating in signaling pathways.^120^ Rezaie et al identified 1030 ncRNAs significantly mutated in breast cancer. They were significantly enriched for eQTL polymorphisms, and their genome-wide association SNP levels were unprecedentedly higher than genome-wide levels.^121^ Recently, Shidal et al conducted a cis-eQTL analysis on the classical breast cancer risk SNP, rs35418111, to re-explore the causative mechanism. The results demonstrated that rs35418111 was associated with reduced expression of the *YBEY* gene, which is involved in a network of genes associated with inflammation and metabolism. Thus, the finding provides an important basis for exploring the association with breast cancer risk.^122^

### Schizophrenia

SZ is a serious mental illness characterized by frequent hallucinations and is caused by changes in brain function. Genetic factors constitute the major factors affecting SZ. eQTL facilitates the identification of genetic markers and the explanation of the complex genetic structure in SZ.^123^ The combination of eQTLs and single-cell RNA sequencing analysis has allowed researchers to deepen their comprehension of SZ further.

In particular, eQTL analysis identified several novel SZ-related loci in the hippocampus and and in cerebrospinal fluid metabolizing species. Yang et al first identified *ALMS1*, *GLT8D1*, and *CSNK2B* as pathophysiologically relevant risk genes in SZ by comprehensive eQTL analysis in the brain tissue.^79^ Another study further confirmed *ALMS1* as a causal gene for SZ.^124^ In addition, Walker et al used a combination of cis-eQTLs and GWAS to identify dozens of novel candidate risk genes, including *SNX19*, *VSP29*, and *XRCC3*.^125^ Schulz et al identified 288 eQTLs enriched in the hippocampus, suggesting the role of the hippocampus in the mechanism of SZ disease.^126^ A cerebrospinal fluid analysis of 414 subjects conducted by Luykx et al revealed that MM-associated eQTL rs11628551 regulates the expression of *PDE9A*, involved in the transmission of monoaminergic signals, as well as major depressive disorder and antidepressant responses.^127^

A combined analysis of the Bayesian statistical framework and Mendelian randomization identified *TYW5* as an SZ risk gene and demonstrated that rs203772 was associated with a high expression of *TYW5* in the prefrontal cortex in the eQTL analysis.^128^ A novel risk missense variant, rs1801311, was identified in 2021 to potentially contribute to SZ risk by regulating *NAGA* expression, causing the up-regulation of *NAGA* expression, and affecting the proliferation and differentiation of neural stem cells.^129^ Furthermore, rs1801311 contributed to SZ by preventing the binding of *YY1*, *TAF1*, and *POLR2A*, requiring further in-depth studies to explore the mechanism. In addition to tissue-derived eQTL, integrated cell-type-based analysis better elucidates the SZ risk genes. Wu et al identified 6108 cis-eQTL genes screened from eight types of brain cells and revealed nine SZ risk genes by analyzing eQTLs in inhibitory neurons.^130^

## Discussion

eQTL as a bridge from the genome to the mechanism, allows investigation of the potential effects between genetic variations and complex traits. In this review, we provided an overview of eQTLs, including their development process, identification and analysis methods, data resources, and their contributions to four typical diseases.

The development of sequencing technology has generated abundant eQTL data, such that the research focus has changed from eQTL identification to its application. eQTL analysis can comprehensively explain the regulatory mechanisms involved in genomic markers, and therefore, it can be performed in multiple ways. For example, eQTL analysis can study the causal association between risk SNPs and phenotypes after GWAS. In addition, it can compare the regulatory differences in eQTLs in case–control samples. eQTL analysis can be combined with other omics data to perform network analysis and infer the regulatory relationships of multiple molecular biomarkers. The integrated analysis of eQTL and single-cell data can provide new clues to uncover the regulatory patterns underlying genetic variations in specific cell types.

Hundreds of eQTLs have been identified in several complex diseases to address the limitations of GWAS and allow causal inference between risk loci and diseases. However, eQTL analysis has certain limitations. Firstly, it is difficult to obtain SNP genotype and gene expression data from the same samples to conduct an eQTL analysis in public databases. Secondly, eQTLs identified in public databases could have regulatory differences in different populations (*e.g.*, cases and controls), which need to be further detected. Thirdly, more efficient tools are required to integrate eQTL and other omics data effectively.

The development of eQTL research has led to the application of its related theories and methods in the research between genetic variations and other biological elements. At least 30 other types of molecular QTLs have emerged, such as protein QTLs influencing protein abundance, methylation QTLs affecting DNA methylation patterns, splicing QTLs regulating alternative transcript isoforms, and metabolic QTLs modulating metabolite levels. While eQTLs primarily capture transcriptional regulation, these complementary QTL classes often map to distinct genomic loci and reveal post-transcriptional, epigenetic, or translational regulatory mechanisms. Notably, partial overlaps between eQTLs and other QTL types may occur when genetic variants exert pleiotropic effects across molecular layers. Integration of multi-omic QTL data enhances our capacity to construct causal regulatory networks and decipher the hierarchical relationships between DNA variation, molecular phenotypes, and organismal traits.^131^^,^^132^ This systematic approach helps disentangle whether the observed expression changes drive downstream biological effects or merely correlate with other molecular events.

Spatial transcriptomics has revolutionized our ability to map gene expression patterns within intact tissue architectures, revealing how cellular microenvironments and spatial organization influence transcriptional regulation.^133^ In this framework, eQTLs, genetic variants associated with gene expression variation, may exhibit spatial context-dependent effects that are obscured in bulk or single-cell RNA-seq analyses. For instance, spatially resolved eQTLs could reflect cell-type-specific regulatory mechanisms localized to distinct tissue regions (*e.g.*, tumor margins *vs*. core) or interactions with spatially varying factors such as hypoxia gradients or immune cell infiltration. Recent studies^134^, ^135^, ^136^ have identified spatial eQTLs, where genetic effects on gene expression are modulated by positional information, suggesting that regulatory genetic variants may drive expression heterogeneity across tissue compartments. Integrating spatial transcriptomics with eQTL mapping could thus disentangle whether genetic regulation operates uniformly or is constrained by niche-specific molecular or cellular interactions. While technical challenges, including limited spatial resolution and statistical power, remain, advances in spatial omics and multivariate analytical frameworks (*e.g.*, tensor-based QTL mapping) promise to uncover how genetic variation shapes spatially organized gene networks.

eQTL studies have emerged as a cornerstone in deciphering the genetic architecture of gene regulation, bridging the gap between genomic variation and phenotypic diversity. Integrating eQTLs with GWAS data enables biomarker discovery and polygenic risk scoring, accelerating precision medicine. The comprehensive integration framework of eQTL and other omics data will bring a new perspective to the understanding of functional genomics in the future. sc-eQTL mapping resolves cellular heterogeneity, capturing transient or context-dependent regulatory effects obscured in bulk analyses. Innovations in scRNA-seq design, such as enriched rare-cell sequencing, enhance the detection of cell-type-specific eQTLs.^65^^,^^90^ These advancements are pivotal for unraveling dynamic processes like immune responses and neuronal activation. Systematic analyses of lncRNA eQTLs will help uncover the potential of non-coding genomics in disease etiology. To sum up, the integration of cutting-edge technologies, diverse population datasets, and multi-omics approaches is revolutionizing eQTL research. These developments not only refine our understanding of genetic regulation but also pave the way for actionable insights into complex diseases, from cancer to neuropsychiatric disorders. As methodologies evolve and resources expand, eQTL studies will play a key role in unlocking the functional genome and delivering tailored therapeutic interventions.

## CRediT authorship contribution statement

**Zhe Jia:** Writing – original draft. **Jing Xu:** Writing – original draft. **Yingnan Ma:** Writing – review & editing. **Siyu Wei:** Writing – review & editing. **Chen Sun:** Software, Resources. **Xingyu Chen:** Software, Conceptualization. **Jingxuan Kang:** Visualization, Conceptualization. **Haiyan Chen:** Methodology, Data curation. **Chen Zhang:** Conceptualization, Formal analysis. **Yu Dong:** Software, Resources. **Junxian Tao:** Resources. **Xuying Guo:** Data curation. **Hongchao Lv:** Supervision, Data curation. **Guoping Tang:** Supervision, Writing – review & editing. **Yongshuai Jiang:** Writing – review & editing, Supervision, Funding acquisition. **Mingming Zhang:** Methodology, Writing – review & editing, Supervision, Funding acquisition.

## Funding

This work was supported by the Program for Young Talents of Basic Research in Universities of Heilongjiang Province, China (No. YQJH2023036), the 10.13039/501100001809National Natural Science Foundation of China (No. 31970651, 92046018, 81601422, 81600403), the Mathematical Tianyuan Fund of the National Natural Science Foundation of China (No. 12026414), the Joint Funds of the Zhejiang Provincial Natural Science Foundation of China (No. LBY24H170001), and the Marshal Initiative Funding of Harbin Medical University, Heilongjiang, China (No. HMUMIF-22010).

## Conflict of interests

The authors declare that they have no known competing financial interests or personal relationships that could have appeared to influence the work reported in this paper.
