# Supplementary material for: eQTL analysis: A bridge from genome to mechanism
Source: Genes Dis. 2025 Sep 17;13(3):101850. doi: 10.1016/j.gendis.2025.101850 (PMC12860985; doi:10.1016/j.gendis.2025.101850)
Supplement: Multimedia component 2 [file mmc2.doc]

Table S4 Information on 4 disease-related risk eQTL genes from whole blood tissue (**P _GTEx_** <1E-15).

| **DISEASE** | **CHR_POS** | **SNPS** | **ENSEMBLE_ID** | **GENE_NAME** | **TSS_DISTANCE** | **P _GTEx_** | **SLOPE_GTEx_** | **P_GWAS_** |
| --- | --- | --- | --- | --- | --- | --- | --- | --- |
| RA | 12_56001170 | rs773125 | ENSG00000197728.9 | RPS26 | -40683 | 6.84E-45 | 0.945059 | 1.00E-09 |
|  | 9_120905921 | rs10435844 | ENSG00000056558.10 | TRAF1 | -23252 | 2.55E-23 | 0.430152 | 1.00E-10 |
|  | 9_120933004 | rs10739580 | ENSG00000056558.10 | TRAF1 | 3831 | 6.59E-23 | 0.429169 | 2.00E-06 |
|  | 9_120944104 | rs2900180 | ENSG00000056558.10 | TRAF1 | 14931 | 6.59E-23 | 0.429169 | 6.00E-09 |
|  | 9_120890620 | rs881375 | ENSG00000056558.10 | TRAF1 | -38553 | 1.69E-22 | 0.422898 | 4.00E-08 |
|  | 6_32614873 | rs3104413 | ENSG00000237541.3 | HLA-DQA2 | -126469 | 1.50E-20 | 1.12207 | 2.00E-10 |
|  | 6_32615580 | rs6931277 | ENSG00000237541.3 | HLA-DQA2 | -125762 | 1.50E-20 | 1.12207 | 2.00E-10 |
|  | 6_32629245 | rs3129769 | ENSG00000237541.3 | HLA-DQA2 | -112097 | 1.50E-20 | 1.12207 | 3.00E-11 |
|  | 6_32460995 | rs9268839 | ENSG00000229391.7 | HLA-DRB6 | -99027 | 1.72E-19 | 0.655359 | 1.00E-250 |
|  | 6_32609603 | rs660895 | ENSG00000237541.3 | HLA-DQA2 | -131739 | 1.78E-19 | 0.989413 | 1.00E-300 |
|  | 6_32476421 | rs12194148 | ENSG00000229391.7 | HLA-DRB6 | -83601 | 4.97E-19 | 0.653234 | 5.00E-58 |
|  | 6_32615965 | rs9271348 | ENSG00000198502.5 | HLA-DRB5 | 85678 | 2.59E-16 | -0.771787 | 5.00E-07 |
|  | 6_32533367 | rs2157337 | ENSG00000198502.5 | HLA-DRB5 | 3080 | 3.20E-16 | -0.65051 | 9.00E-52 |
|  | 15_90350436 | rs6496667 | ENSG00000140575.12 | IQGAP1 | -37782 | 4.73E-16 | 0.418372 | 1.00E-06 |

Continuation of Table S4

| **DISEASE** | **CHR_POS** | **SNPS** | **ENSEMBLE_ID** | **GENE_NAME** | **TSS_DISTANCE** | **P _GTEx_** | **SLOPE_GTEx_** | **P_GWAS_** |
| --- | --- | --- | --- | --- | --- | --- | --- | --- |
| T2D | 12_56076841 | rs11171739 | ENSG00000197728.9 | RPS26 | 34988 | 2.76E-70 | -1.05239 | 1.00E-18 |
|  | 7_55734370 | rs6972291 | ENSG00000226278.1 | PSPHP1 | -30427 | 6.88E-51 | 1.20974 | 2.00E-09 |
|  | 11_43856384 | rs1061810 | ENSG00000149084.12 | HSD17B12 | 175704 | 2.57E-43 | -0.733543 | 6.00E-13 |
|  | 11_43856909 | rs35251247 | ENSG00000149084.12 | HSD17B12 | 176229 | 9.70E-43 | -0.730283 | 2.00E-13 |
|  | 17_50559173 | rs989128 | ENSG00000006282.20 | SPATA20 | 12867 | 1.42E-37 | -0.833929 | 4.00E-06 |
|  | 6_32659937 | rs1063355 | ENSG00000179344.16 | HLA-DQB1 | -8446 | 3.02E-37 | -0.804956 | 4.00E-19 |
|  | 3_53093661 | rs2581787 | ENSG00000242142.1 | SERBP1P3 | 28570 | 6.88E-37 | 0.85084 | 3.00E-08 |
|  | 6_32659937 | rs1063355 | ENSG00000232629.8 | HLA-DQB2 | -103597 | 1.23E-29 | 0.84918 | 4.00E-19 |
|  | 10_100216744 | rs11591741 | ENSG00000095485.16 | CWF19L1 | -50936 | 1.06E-28 | 0.801112 | 1.00E-09 |
|  | 16_3584745 | rs12933120 | ENSG00000103351.12 | CLUAP1 | 83821 | 2.04E-27 | 1.04875 | 3.00E-10 |
|  | 16_53492639 | rs9931702 | ENSG00000103479.15 | RBL2 | 58662 | 1.03E-26 | -0.406794 | 4.00E-08 |
|  | 5_56808481 | rs10461617 | ENSG00000155542.11 | SETD9 | -100779 | 1.09E-26 | 1.04249 | 4.00E-06 |
|  | 11_49889289 | rs1794138 | ENSG00000255190.2 | TRIM51DP | 14344 | 2.27E-26 | 0.853605 | 7.00E-09 |
|  | 6_32626532 | rs9271774 | ENSG00000198502.5 | HLA-DRB5 | 96245 | 3.81E-25 | -0.903004 | 1.00E-09 |

Continuation of Table S4

| **DISEASE** | **CHR_POS** | **SNPS** | **ENSEMBLE_ID** | **GENE_NAME** | **TSS_DISTANCE** | **P _GTEx_** | **SLOPE_GTEx_** | **P_GWAS_** |
| --- | --- | --- | --- | --- | --- | --- | --- | --- |
| T2D | 16_53468034 | rs4281707 | ENSG00000103479.15 | RBL2 | 34057 | 3.86E-25 | -0.381477 | 3.00E-10 |
|  | 7_55734370 | rs6972291 | ENSG00000185290.3 | NUPR2 | -382030 | 9.50E-24 | 0.843069 | 2.00E-09 |
|  | 6_125731213 | rs2008027 | ENSG00000237742.6 | HEY2-AS1 | -17977 | 1.73E-23 | -0.622885 | 4.00E-09 |
|  | 6_125740356 | rs7758115 | ENSG00000237742.6 | HEY2-AS1 | -8834 | 1.73E-23 | -0.622885 | 4.00E-09 |
|  | 11_119082492 | rs7127212 | ENSG00000160695.14 | VPS11 | 14793 | 2.18E-23 | -0.618995 | 2.00E-08 |
|  | 3_49958085 | rs6792892 | ENSG00000004534.14 | RBM6 | 18078 | 1.23E-22 | -0.382712 | 1.00E-14 |
|  | 10_100152307 | rs2862954 | ENSG00000095485.16 | CWF19L1 | -115373 | 2.21E-22 | 0.710329 | 2.00E-10 |
|  | 10_100152437 | rs1408579 | ENSG00000095485.16 | CWF19L1 | -115243 | 2.21E-22 | 0.710329 | 6.00E-11 |
|  | 20_38392336 | rs736368 | ENSG00000196756.11 | SNHG17 | -43017 | 2.81E-22 | 1.00517 | 9.00E-06 |
|  | 17_46006582 | rs8067056 | ENSG00000214425.7 | LRRC37A4P | 456247 | 4.93E-22 | -0.71987 | 3.00E-08 |
|  | 3_53093661 | rs2581787 | ENSG00000163933.9 | RFT1 | -36801 | 1.84E-20 | -0.737741 | 3.00E-08 |
|  | 3_53093661 | rs2581787 | ENSG00000163933.9 | RFT1 | -36801 | 1.84E-20 | -0.737741 | 2.00E-08 |
|  | 7_74674569 | rs35473599 | ENSG00000196275.13 | GTF2IRD2 | -176982 | 8.63E-20 | -0.680411 | 3.00E-09 |
|  | 6_32659937 | rs1063355 | ENSG00000237541.3 | HLA-DQA2 | -81405 | 1.08E-19 | 0.7854 | 4.00E-19 |

Continuation of Table S4

| **DISEASE** | **CHR_POS** | **SNPS** | **ENSEMBLE_ID** | **GENE_NAME** | **TSS_DISTANCE** | **P _GTEx_** | **SLOPE_GTEx_** | **P_GWAS_** |
| --- | --- | --- | --- | --- | --- | --- | --- | --- |
| T2D | 17_78765957 | rs62075585 | ENSG00000055483.19 | USP36 | -75484 | 2.26E-19 | 0.465229 | 1.00E-10 |
|  | 16_3584745 | rs12933120 | ENSG00000167984.17 | NLRC3 | 7345 | 2.69E-19 | -1.08448 | 3.00E-10 |
|  | 6_32605638 | rs601945 | ENSG00000237541.3 | HLA-DQA2 | -135704 | 4.10E-19 | 1.12853 | 5.00E-08 |
|  | 6_32626532 | rs9271774 | ENSG00000229391.7 | HLA-DRB6 | 66510 | 4.91E-19 | 0.754492 | 1.00E-09 |
|  | 6_32460338 | rs115918645 | ENSG00000229391.7 | HLA-DRB6 | -99684 | 7.51E-19 | 0.753159 | 1.00E-11 |
|  | 6_32460338 | rs115918645 | ENSG00000237541.3 | HLA-DQA2 | -281004 | 9.87E-19 | 0.90486 | 1.00E-11 |
|  | 16_3597097 | rs2240885 | ENSG00000103351.12 | CLUAP1 | 96173 | 9.93E-19 | 0.718291 | 3.00E-09 |
|  | 16_3533173 | rs3751837 | ENSG00000167984.17 | NLRC3 | -44227 | 1.26E-18 | -0.84982 | 1.00E-08 |
|  | 15_75522047 | rs6495182 | ENSG00000140400.16 | MAN2C1 | 153893 | 1.96E-18 | 0.42319 | 2.00E-22 |
|  | 16_3606481 | rs8061528 | ENSG00000103351.12 | CLUAP1 | 105557 | 3.06E-18 | 0.756997 | 3.00E-14 |
|  | 16_3533173 | rs3751837 | ENSG00000103351.12 | CLUAP1 | 32249 | 5.30E-18 | 0.707781 | 1.00E-08 |
|  | 15_75449754 | rs8038760 | ENSG00000140400.16 | MAN2C1 | 81600 | 1.35E-17 | 0.410918 | 2.00E-11 |
|  | 15_90979023 | rs8026714 | ENSG00000166965.12 | RCCD1 | 23227 | 1.69E-17 | 0.793469 | 1.00E-22 |
|  | 15_43602920 | rs2447198 | ENSG00000168803.14 | ADAL | 272246 | 1.81E-17 | -0.902114 | 5.00E-10 |

Continuation of Table S4

| **DISEASE** | **CHR_POS** | **SNPS** | **ENSEMBLE_ID** | **GENE_NAME** | **TSS_DISTANCE** | **P _GTEx_** | **SLOPE_GTEx_** | **P_GWAS_** |
| --- | --- | --- | --- | --- | --- | --- | --- | --- |
| T2D | 19_12927601 | rs3111316 | ENSG00000105607.12 | GCDH | 36575 | 2.01E-17 | -0.422996 | 3.00E-21 |
|  | 15_90962549 | rs79548680 | ENSG00000166965.12 | RCCD1 | 6753 | 2.10E-17 | 0.802136 | 4.00E-06 |
|  | 7_74693803 | rs67755137 | ENSG00000196275.13 | GTF2IRD2 | -157748 | 3.16E-17 | -0.716469 | 2.00E-08 |
|  | 17_78776206 | rs7224711 | ENSG00000055483.19 | USP36 | -65235 | 3.62E-17 | 0.427235 | 1.00E-15 |
|  | 6_32170768 | rs3130283 | ENSG00000198502.5 | HLA-DRB5 | -359519 | 4.54E-17 | -0.982963 | 6.00E-27 |
|  | 10_100060908 | rs12782078 | ENSG00000095485.16 | CWF19L1 | -206772 | 9.07E-17 | 0.640736 | 2.00E-08 |
|  | 12_56049558 | rs11171731 | ENSG00000197728.9 | RPS26 | 7705 | 2.21E-16 | -0.691682 | 9.00E-06 |
|  | 7_7210116 | rs11763876 | ENSG00000106392.10 | C1GALT1 | 53182 | 3.47E-16 | 0.815193 | 7.00E-08 |
|  | 17_46006582 | rs8067056 | ENSG00000204650.14 | LINC02210 | 386254 | 3.85E-16 | 0.585732 | 3.00E-08 |
|  | 6_32634705 | rs3104369 | ENSG00000229391.7 | HLA-DRB6 | 74683 | 4.53E-16 | 0.674164 | 9.00E-15 |
|  | 6_32634705 | rs3104369 | ENSG00000179344.16 | HLA-DQB1 | -33678 | 4.93E-16 | -0.663162 | 9.00E-15 |
|  | 17_48889676 | rs79349575 | ENSG00000248278.1 | SUMO2P17 | -19307 | 6.64E-16 | 0.785648 | 4.00E-08 |
|  | 17_45941122 | rs62061734 | ENSG00000214425.7 | LRRC37A4P | 390787 | 9.48E-43 | -1.00563 | 8.00E-12 |
| BC | 17_46751565 | rs199533 | ENSG00000214401.4 | KANSL1-AS1 | 557989 | 4.12E-42 | 1.17389 | 5.00E-08 |

Continuation of Table S4

| **DISEASE** | **CHR_POS** | **SNPS** | **ENSEMBLE_ID** | **GENE_NAME** | **TSS_DISTANCE** | **P _GTEx_** | **SLOPE_GTEx_** | **P_GWAS_** |
| --- | --- | --- | --- | --- | --- | --- | --- | --- |
| BC | 17_46751565 | rs199533 | ENSG00000238083.7 | LRRC37A2 | 240054 | 4.23E-38 | 1.17802 | 5.00E-08 |
|  | 17_45941122 | rs62061734 | ENSG00000238083.7 | LRRC37A2 | -570389 | 5.61E-36 | 1.18858 | 8.00E-12 |
|  | 6_33272092 | rs17215231 | ENSG00000231500.6 | RPS18 | 44 | 1.14E-32 | -0.780408 | 9.00E-13 |
|  | 17_46751565 | rs199533 | ENSG00000262500.1 | MAPK8IP1P1 | 507959 | 1.43E-32 | 1.14671 | 5.00E-08 |
|  | 17_45941122 | rs62061734 | ENSG00000214401.4 | KANSL1-AS1 | -252454 | 2.45E-32 | 1.114 | 8.00E-12 |
|  | 17_45941122 | rs62061734 | ENSG00000204650.14 | LINC02210 | 320794 | 4.75E-31 | 0.846779 | 8.00E-12 |
|  | 17_45941122 | rs62061734 | ENSG00000262500.1 | MAPK8IP1P1 | -302484 | 2.45E-30 | 1.14964 | 8.00E-12 |
|  | 9_104094512 | rs4742903 | ENSG00000136824.18 | SMC2 | 252 | 1.97E-28 | -0.672856 | 2.00E-07 |
|  | 5_82242227 | rs7707921 | ENSG00000186468.12 | RPS23 | -36350 | 1.59E-27 | 0.690197 | 4.00E-09 |
|  | 17_46788237 | rs199498 | ENSG00000238083.7 | LRRC37A2 | 276726 | 1.52E-26 | 1.08994 | 2.00E-08 |
|  | 5_82161539 | rs2407064 | ENSG00000186468.12 | RPS23 | -117038 | 1.57E-26 | 0.687136 | 3.00E-08 |
|  | 5_81972251 | rs111549985 | ENSG00000186468.12 | RPS23 | -306326 | 5.64E-26 | -0.682031 | 6.00E-12 |
|  | 17_46788237 | rs199498 | ENSG00000214401.4 | KANSL1-AS1 | 594661 | 3.59E-25 | 1.03591 | 2.00E-08 |
|  | 17_45394123 | rs4763 | ENSG00000214425.7 | LRRC37A4P | -156212 | 2.23E-24 | -0.887507 | 2.00E-08 |

Continuation of Table S4

| **DISEASE** | **CHR_POS** | **SNPS** | **ENSEMBLE_ID** | **GENE_NAME** | **TSS_DISTANCE** | **P _GTEx_** | **SLOPE_GTEx_** | **P_GWAS_** |
| --- | --- | --- | --- | --- | --- | --- | --- | --- |
| BC | 17_45941122 | rs62061734 | ENSG00000263503.1 | MAPK8IP1P2 | 338782 | 2.32E-24 | 1.0743 | 8.00E-12 |
|  | 2_201288355 | rs1035142 | ENSG00000155749.12 | FLACC1 | -69043 | 7.39E-24 | -0.474304 | 3.00E-09 |
|  | 17_45941122 | rs62061734 | ENSG00000264070.1 | DND1P1 | 355251 | 1.56E-23 | 1.02003 | 8.00E-12 |
|  | 11_803017 | rs6597981 | ENSG00000255284.1 | GATD1-DT | 25439 | 2.42E-23 | 0.762731 | 1.00E-12 |
|  | 5_82242227 | rs7707921 | ENSG00000205464.11 | ATP6AP1L | -37235 | 6.87E-23 | -0.691636 | 4.00E-09 |
|  | 17_46788237 | rs199498 | ENSG00000262500.1 | MAPK8IP1P1 | 544631 | 1.70E-22 | 1.04622 | 2.00E-08 |
|  | 5_82340549 | rs2407156 | ENSG00000186468.12 | RPS23 | 61972 | 4.49E-22 | -0.592539 | 2.00E-06 |
|  | 5_82340549 | rs2407156 | ENSG00000205464.11 | ATP6AP1L | 61087 | 4.13E-20 | 0.612441 | 2.00E-06 |
|  | 15_90965985 | rs77554484 | ENSG00000166965.12 | RCCD1 | 10189 | 5.09E-20 | 0.891414 | 1.00E-15 |
|  | 17_45394123 | rs4763 | ENSG00000204650.14 | LINC02210 | -226205 | 2.62E-19 | 0.749978 | 2.00E-08 |
|  | 19_43919418 | rs375066 | ENSG00000176222.8 | ZNF404 | 35367 | 1.49E-18 | -0.552121 | 2.00E-14 |
|  | 5_81972251 | rs111549985 | ENSG00000205464.11 | ATP6AP1L | -307211 | 1.51E-18 | 0.641102 | 6.00E-12 |
|  | 17_45394123 | rs4763 | ENSG00000214401.4 | KANSL1-AS1 | -799453 | 3.61E-17 | 0.926481 | 2.00E-08 |
|  | 17_46751565 | rs199533 | ENSG00000185829.17 | ARL17A | 171883 | 1.02E-16 | 0.827488 | 5.00E-08 |

Continuation of Table S4

| **DISEASE** | **CHR_POS** | **SNPS** | **ENSEMBLE_ID** | **GENE_NAME** | **TSS_DISTANCE** | **P _GTEx_** | **SLOPE_GTEx_** | **P_GWAS_** |
| --- | --- | --- | --- | --- | --- | --- | --- | --- |
| BC | 17_45394123 | rs4763 | ENSG00000262500.1 | MAPK8IP1P1 | -849483 | 2.90E-16 | 0.953632 | 2.00E-08 |
|  | 17_45941122 | rs62061734 | ENSG00000185829.17 | ARL17A | -638560 | 4.62E-16 | 0.834403 | 8.00E-12 |
| SZ | 18_12947463 | rs3809912 | ENSG00000101639.18 | CEP192 | -43899 | 4.87E-43 | -0.925836 | 1.00E-08 |
|  | 6_28195002 | rs1150688 | ENSG00000216901.1 | ZNF603P | 18814 | 3.34E-42 | 1.14016 | 8.00E-11 |
|  | 17_45666978 | rs1635298 | ENSG00000214425.7 | LRRC37A4P | 116643 | 1.35E-37 | 0.895483 | 3.00E-08 |
|  | 22_42141587 | rs1800754 | ENSG00000100197.20 | CYP2D6 | 10681 | 5.62E-36 | 0.762642 | 2.00E-15 |
|  | 17_46784796 | rs199503 | ENSG00000214401.4 | KANSL1-AS1 | 591220 | 1.12E-35 | 1.1461 | 3.00E-09 |
|  | 12_56055651 | rs7302200 | ENSG00000197728.9 | RPS26 | 13798 | 4.62E-34 | 0.947648 | 1.00E-14 |
|  | 17_46784796 | rs199503 | ENSG00000238083.7 | LRRC37A2 | 273285 | 3.08E-32 | 1.1458 | 3.00E-09 |
|  | 17_45666978 | rs1635298 | ENSG00000238083.7 | LRRC37A2 | -844533 | 1.04E-30 | -1.04332 | 3.00E-08 |
|  | 17_45666978 | rs1635298 | ENSG00000214401.4 | KANSL1-AS1 | -526598 | 1.29E-30 | -1.00993 | 3.00E-08 |
|  | 17_46784796 | rs199503 | ENSG00000262500.1 | MAPK8IP1P1 | 541190 | 2.12E-30 | 1.14773 | 3.00E-09 |
|  | 17_45666978 | rs1635298 | ENSG00000204650.14 | LINC02210 | 46650 | 3.07E-29 | -0.765963 | 3.00E-08 |
|  | 16_11983775 | rs12922317 | ENSG00000234719.8 | NPIPB2 | 7132 | 1.41E-28 | 1.06106 | 9.00E-07 |

Continuation of Table S4

| **DISEASE** | **CHR_POS** | **SNPS** | **ENSEMBLE_ID** | **GENE_NAME** | **TSS_DISTANCE** | **P _GTEx_** | **SLOPE_GTEx_** | **P_GWAS_** |
| --- | --- | --- | --- | --- | --- | --- | --- | --- |
| SZ | 11_49681564 | rs11040472 | ENSG00000255190.2 | TRIM51DP | -193381 | 5.15E-27 | 0.872688 | 3.00E-06 |
|  | 17_45666978 | rs1635298 | ENSG00000262500.1 | MAPK8IP1P1 | -576628 | 2.86E-24 | -0.980192 | 3.00E-08 |
|  | 10_100152437 | rs1408579 | ENSG00000095485.16 | CWF19L1 | -115243 | 2.21E-22 | 0.710329 | 2.00E-08 |
|  | 17_45666978 | rs1635298 | ENSG00000263503.1 | MAPK8IP1P2 | 64638 | 1.49E-21 | -0.945883 | 3.00E-08 |
|  | 17_45666978 | rs1635298 | ENSG00000264070.1 | DND1P1 | 81107 | 1.66E-21 | -0.909138 | 3.00E-08 |
|  | 8_8240516 | rs2945232 | ENSG00000253893.2 | FAM85B | 13902 | 2.03E-21 | -0.835289 | 2.00E-08 |
|  | 3_50002761 | rs4688756 | ENSG00000004534.14 | RBM6 | 62754 | 2.28E-21 | -0.373737 | 2.00E-17 |
|  | 17_12993236 | rs9908102 | ENSG00000006744.18 | ELAC2 | -24951 | 8.21E-21 | 0.528471 | 2.00E-06 |
|  | 6_29972902 | rs114204022 | ENSG00000230795.3 | HLA-K | 45724 | 3.89E-20 | -0.874244 | 5.00E-11 |
|  | 2_48153579 | rs12475492 | ENSG00000170802.15 | FOXN2 | -161058 | 8.06E-20 | 0.463521 | 6.00E-09 |
|  | 10_102852578 | rs11191419 | ENSG00000166275.15 | BORCS7 | -1645 | 2.89E-19 | -0.502666 | 3.00E-18 |
|  | 8_8234503 | rs2980436 | ENSG00000253893.2 | FAM85B | 7889 | 1.70E-18 | -0.826225 | 4.00E-07 |
|  | 3_52821213 | rs2239547 | ENSG00000055955.15 | ITIH4 | -9526 | 1.79E-18 | 0.581811 | 6.00E-08 |
|  | 12_76927801 | rs7302529 | ENSG00000175183.9 | CSRP2 | 48741 | 2.00E-18 | 0.608832 | 7.00E-07 |

Continuation of Table S4

| **DISEASE** | **CHR_POS** | **SNPS** | **ENSEMBLE_ID** | **GENE_NAME** | **TSS_DISTANCE** | **P _GTEx_** | **SLOPE_GTEx_** | **P_GWAS_** |
| --- | --- | --- | --- | --- | --- | --- | --- | --- |
| SZ | 10_102866129 | rs11191424 | ENSG00000166275.15 | BORCS7 | 11906 | 7.43E-18 | -0.479962 | 4.00E-20 |
|  | 2_47993197 | rs17396122 | ENSG00000170802.15 | FOXN2 | -321440 | 1.33E-17 | 0.451178 | 1.00E-06 |
|  | 8_8234883 | rs1878561 | ENSG00000253893.2 | FAM85B | 8269 | 3.62E-17 | -0.720886 | 1.00E-07 |
|  | 6_32597688 | rs2760981 | ENSG00000198502.5 | HLA-DRB5 | 67401 | 1.16E-16 | -0.683664 | 4.00E-11 |
|  | 17_45779092 | rs35076622 | ENSG00000214425.7 | LRRC37A4P | 228757 | 1.25E-16 | -0.630694 | 3.00E-12 |
|  | 17_45779092 | rs35076622 | ENSG00000214401.4 | KANSL1-AS1 | -414484 | 1.66E-16 | 0.756127 | 3.00E-12 |
|  | 6_32668836 | rs147875011 | ENSG00000232629.8 | HLA-DQB2 | -94698 | 2.05E-16 | 0.670291 | 3.00E-10 |
|  | 6_32668836 | rs147875011 | ENSG00000179344.16 | HLA-DQB1 | 453 | 4.63E-16 | -0.585269 | 3.00E-10 |
|  | 14_22912771 | rs9989230 | ENSG00000092036.18 | HAUS4 | -44390 | 4.71E-16 | -0.424041 | 4.00E-12 |
|  | 17_46784796 | rs199503 | ENSG00000185829.17 | ARL17A | 205114 | 5.04E-16 | 0.831552 | 3.00E-09 |
|  | 14_22912771 | rs9989230 | ENSG00000257285.5 | PRMT5-DT | -16838 | 8.65E-16 | 0.708601 | 4.00E-12 |
|  | 17_45666978 | rs1635298 | ENSG00000185829.17 | ARL17A | -912704 | 9.93E-16 | -0.763039 | 3.00E-08 |
